# Regenerating the liver: not so simple after all?

**DOI:** 10.12688/f1000research.8827.1

**Published:** 2016-07-26

**Authors:** Malcolm R. Alison, Wey-Ran Lin

**Affiliations:** 1 Centre for Tumour Biology, Barts and The London School of Medicine and Dentistry, London, UK; 2 Department of Gastroenterology and Hepatology, Linkou Chang Gung Memorial Hospital, Taoyuan 333, Taiwan; 3 Department of Medicine, Chang Gung University, Taoyuan 333, Taiwan

**Keywords:** hepatocyte renewal, hepatocyte regeneration, pericentral hepatocytes, periportal hepatocytes, liver regeneration

## Abstract

Under normal homeostatic conditions, hepatocyte renewal is a slow process and complete turnover likely takes at least a year. Studies of hepatocyte regeneration after a two-thirds partial hepatectomy (2/3 PH) have strongly suggested that periportal hepatocytes are the driving force behind regenerative re-population, but recent murine studies have brought greater complexity to the issue. Although periportal hepatocytes are still considered pre-eminent in the response to 2/3 PH, new studies suggest that normal homeostatic renewal is driven by pericentral hepatocytes under the control of Wnts, while pericentral injury provokes the clonal expansion of a subpopulation of periportal hepatocytes expressing low levels of biliary duct genes such as *Sox9* and *osteopontin*. Furthermore, some clarity has been given to the debate on the ability of biliary-derived hepatic progenitor cells to generate physiologically meaningful numbers of hepatocytes in injury models, demonstrating that under appropriate circumstances these cells can re-populate the whole liver.

## Introduction

Liver disease is an increasing problem worldwide, and with the seemingly intractable problem of an insufficient number of livers available for transplantation, attention is turning to alternate sources of supply. In particular, the ability to *ex vivo*-expand the number of good-quality hepatocytes from the limited number of available livers is viewed as an attractive proposition. Thus, much effort is being spent in defining liver populations in terms of their ‘stemness’ or clonogenic potential, cell surface characteristics (for isolation), and their location within the liver. Although our understanding of the organization and renewal of the likes of the hematopoietic system and the small intestine is well advanced, it might surprise readers of this article to find that hepatologists cannot even agree whether the liver has a hierarchical organization, conforming to a stem cell and lineage system.

Many previous studies into the kinetics of hepatocyte proliferation have concentrated on the periportal zone, since hepatocytes located here are the first to enter DNA synthesis after a two-thirds partial hepatectomy (2/3 PH) (reviewed in 1) and undergo more rounds of replication than midzonal and pericentral hepatocytes. In the ‘80s, pulse-chase analysis of rats injected with tritiated thymidine suggested a slow migration of hepatocytes along the portal vein (PV)-to-central vein (CV) axis, formally proposed as the ‘streaming liver’ hypothesis ^2^. Studies in human liver have also supported a periportal origin of hepatocyte generation; distinct maturational stages were found along the same PV-CV axis ^3^, and while studying mitochondrial DNA (mtDNA) mutation analysis, we have discovered clonal populations of hepatocytes invariably connected to the portal limiting plate ^4, 5^.

The long-term retention of DNA labels in cells has been advanced as evidence for slowly cycling stem cells, and in the mouse DNA label-retaining cells are in and around the portal tracts, providing further support for a periportal stem cell niche in this location ^6^. Employing tamoxifen-inducible genetic lineage tracing from the *Sox9* locus in the mouse liver, Furuyama *et al*. ^7^ followed the fate of labeled biliary cells by X-gal staining, observing marked cells seemingly migrating along the PV-CV axis to eventually replace the whole parenchyma within 8 to 12 months. These observations would imply that cells within the biliary tree are drivers not only of hepatocyte replacement when regeneration from existing hepatocytes is compromised—see section on hepatic progenitor cells (HPCs), entitled ‘periportal/portal stem cell niche(s)’—but also of normal hepatocyte turnover. Unfortunately, these findings have not been replicated by others. For example, Tarlow *et al*. ^8^ found that Sox9-positive ductal cells made only a minimal contribution to parenchymal regeneration in a number of liver injury models; moreover, even when high-dose tamoxifen was used to induce Sox9 expression in periportal hepatocytes, these marked hepatocytes did not replace the remainder of the parenchyma. Lineage labeling from Sox9-expressing ductal plate cells also indicated no large-scale parenchymal replacement from these cells, descendants being restricted to a few periportal hepatocytes as well as the intrahepatic biliary tree ^9^. Achieving specific marker gene activation in all mouse hepatocytes with an adeno-associated viral vector has also failed to indicate any significant contribution of biliary cells to the hepatic parenchyma, not only under normal homeostatic conditions but also after 2/3 PH or carbon tetrachloride (CCl _4_) toxic injury ^10^. Retroviral-mediated β-galactosidase gene transfer to rat livers 24 hours after a 2/3 PH has also seemingly put a ‘nail in the coffin’ of the ‘streaming liver’ hypothesis ^11, 12^, given that there was no apparent movement of these marked hepatocytes over the next year or more. A recent review summarizes ^13^ these two studies with the statement “it is important to consider that much literature refutes the notion of either portal-to-central or central-to-portal hepatocyte streaming during homeostasis”. This review now describes several studies that re-invigorate the streaming debate as well as provide convincing evidence that biliary-derived HPCs can be an effective reservoir for new parenchymal (hepatocyte) cells.

## A pericentral hepatic stem cell niche?

**Figure 1.**
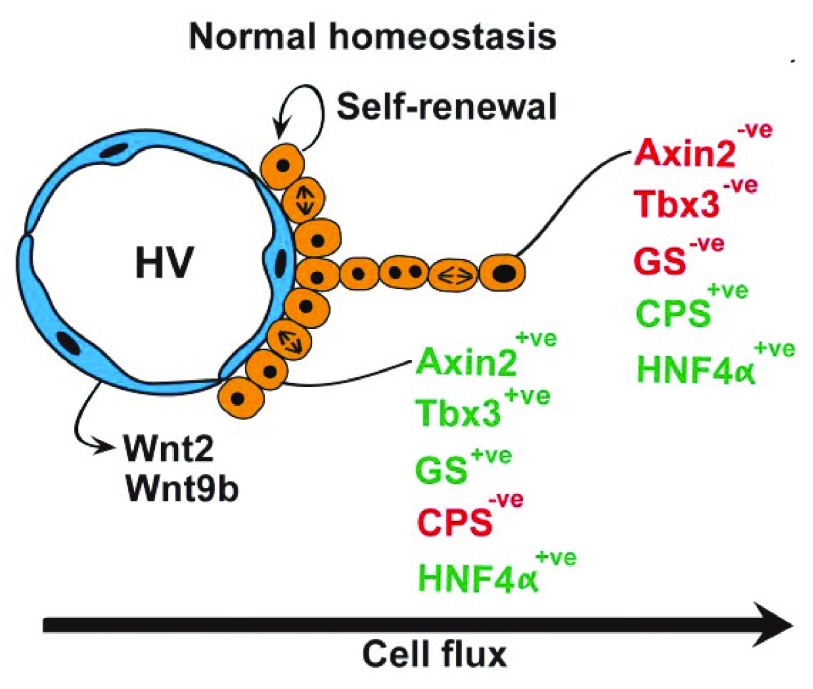
A pericentral stem/progenitor niche. Under normal homeostatic conditions essentially diploid hepatocytes abut the central veins, they may self-renew, and the progeny of these cells migrate concentrically away from the central vein towards the portal regions. This migration is accompanied by polyploidization and changes in metabolic status appropriate to position along the central vein-portal vein axis. See section entitled ‘a pericentral hepatic stem cell niche?’ and 14 for further details.

In the mouse, a single layer of essentially diploid hepatocytes (two-thirds diploid and one-third tetraploid) abuts the hepatic (central) veins, expressing the Wnt target gene products Axin2 (axis inhibition protein 2) and glutamine synthetase (GS) ^14^. Axin2 negatively regulates Wnt signaling, promoting phosphorylation and degradation of β-catenin ^15^. These hepatocytes lack expression of the differentiated gene product carbamoyl-phosphate synthase 1 (CPS-1) but do express the transcription factors HNF4α (for hepatocyte fate determination) and Tbx3 (a pluripotency factor); Tbx is also expressed in hepatoblasts. Under normal homeostatic conditions, genetic lineage labeling from these Axin2-positive hepatocytes with inducible Cre revealed that these cells migrated concentrically away from the hepatic veins, differentiating into Axin2- and GS-negative but CPS-positive polyploid hepatocytes that within a year had reached the portal rim ( Figure 1). No cholangiocyte differentiation was seen from these cells. Hepatic vein endothelial cells expressed high levels of Wnt2 and Wnt9b, and disruption of this Wnt signaling reduced both Axin2 expression and the rate of cell proliferation of these Axin2-positive hepatocytes. Thus, hepatic vein endothelial cells could constitute the stem cell niche. Tracing dilution of a stable DNA label, the authors estimated that the Axin2-positive hepatocytes divided every 14 days, twice as fast as Axin2-negative hepatocytes. Moreover, the pericentral ring of Axin2-positive hepatocytes was never infiltrated by Axin2-negative hepatocytes, suggesting to the authors that the Axin2-positive population is self-renewing—one functional definition of stem cells. On the other hand, Axin2-negative hepatocytes could have penetrated the pericentral hepatocyte ring, becoming Axin2 positive when in contact with the hepatic venous system. Indeed, over 20 years ago, Kuo and Darnell ^16^ noted that widening of the GS-positive zone did not occur after a 75% hepatectomy in mice even though the GS-positive hepatocytes divided, reasoning (correctly, it seems) that the immediate microenvironment of the hepatic venous system provides the signals for GS gene expression.

Wnts regulate stem cell renewal in many tissues, and in mouse liver Wnt signaling is implicated in metabolic zonation ^17^ and liver regeneration ^18^, seemingly spatially coordinated by the combination of R-spondin (RSPO) ligands, their leucine-rich repeat-containing G protein-coupled receptors LGR4 and LGR5 and the ZNRF3/RNF43 receptor-degrading enzymes ^19, 20^. Planas-Paz *et al*. ^19^ found that RSPO1 improved liver regeneration but that LGR4/5 loss of function resulted in hypoplasia; the authors concluded that the RSPO-LGR4/5-ZNRF3/RNF43 module not only controls metabolic zonation but also acts as a hepatic growth/size rheostat. Surprisingly, the authors failed to confirm the observations of the Nusse group ^14^, finding that the LGR5-expressing pericentral hepatocytes did not show an increased proliferative rate compared with hepatocytes in other zones, nor did they find that descendants of these pericentral hepatocytes extensively re-populated the liver during normal homeostasis or following a 2/3 PH! Clearly, further studies are required to clarify the role of pericentral hepatocytes.

Apart from the role of Wnt signaling, many other growth factors, cytokines, and their signaling pathways have been implicated in initiating and terminating hepatocyte proliferation; these have been comprehensively reviewed elsewhere ^1, 21– 24^. Table 1 highlights some of the more recent findings that could have clinical implications ^25– 43^.

**Table 1.** Selected studies related to the regulation of hepatocyte proliferation.

Reference	Observations	Comment
Buitrago-Molina *et al*. (2013) ^25^	Deletion of p21 in mice with severe liver injury leads to continued proliferation and facilitates HCC development.	p21 loss impairs regeneration in mice with chronic moderate injury with upregulation of sestrin-2. Sestrin-2 inhibits mTOR-mediated hepatocyte proliferation but also enhances the Nrf2-regulated oxidative stress response.
Nejak-Bowen *et al*. (2013) ^26^	NF-κB p65/β-catenin complex dissociates after tumor necrosis factor-alpha induced injury. β-catenin KO mice have an earlier, stronger, and more protracted activation of NF-κB than WT mice.	β-catenin inhibition in the context of cancer may have unintended consequences of promoting tumor cell survival.
Xia *et al*. (2013) ^27^	HDAC1 and HDAC2 associate independently with C/EBPβ to upregulate Ki-67 expression.	Loss of HDAC1/2 impairs regeneration.
Xu *et al*. (2013) ^28^	A long non-coding RNA (lncRNA) specifically differentially expressed during liver regeneration facilitates cyclin D1 expression by potentiating Wnt/β- catenin signaling through Axin1 suppression.	Pharmacological targeting of specific lncRNAs may aid regeneration.
Yuan *et al*. (2013) ^29^	miR-221 promotes liver regeneration by targeting p27, p57, and aryl hydrocarbon nuclear translocator (Arnt).	Knockdown of miR-221 in HCC could reduce growth rate.
Amaya *et al*. (2014) ^30^	A subset of insulin receptors localize to the nucleus upon insulin binding, generating InsP _3_-dependent Ca ^2+^ signals with pro-proliferative effects.	A number of potential targets have been identified for modulating hepatocyte proliferation.
Fanti *et al*. (2014) ^31^	Thyroid hormone (T3) promotes β-catenin-TCF4 reporter activity through protein kinase A-dependent β-catenin activation (phosphorylation of Ser675).	T3 may be useful to induce regeneration in cases of hepatic insufficiency.
Garcia-Rodriguez *et al*. (2014) ^32^	Over-expression of sirtuin1 (SIRT1), a class III histone deacetylase, impairs regeneration after PH.	Aberrant SIRT1 over-expression could be targeted in metabolic liver disease involving dysregulated bile acid metabolism.
Kohler *et al*. (2014) ^33^	High levels of activated Nrf2 delay regeneration after PH, possibly allowing time for damage repair before proliferation.	Caution is advised when using Nrf2-activating compounds for the prevention of liver damage.
Rizzo *et al*. (2014) ^34^	Seventy-two out of about 1400 piRNAs show changes in expression 24 to 48 hours after PH, returning to basal levels by 168 hours.	The role of piRNAs in regeneration is unclear but is a new field of investigation.
Starlinger *et al*. (2014) ^35^	Patients with low intraplatelet levels of serotonin (5-HT) have delayed hepatic regeneration.	Platelet levels of serotonin may predict clinical outcome after hepatic resection.
Jin *et al*. (2015) ^36^	Expression of C/EBPα opposes the pro-proliferative effects of C/EBPβ. Absence of active C/EBPα leads to hepatomegaly.	Deregulation of the formation of complexes between C/EBP proteins and chromatin remodeling proteins disrupts liver homeostasis.
Nguyen *et al*. (2015) ^37^	YAP is a powerful stimulant of hepatic growth that can be degraded via phosphorylation by the Hippo signaling pathway.	Reducing YAP protein levels or targeting YAP-TEAD interactions may reduce hepatocyte/biliary proliferation.
Yang and Monga (2015) ^38^	Hepatocyte-secreted Wnt5a suppresses β-catenin signaling in an autocrine manner through Frizzled-2, terminating regeneration.	Loss of termination signals such as Wnt5a may contribute to dysregulated growth (for example, in tumors).
Zhang *et al*. (2015) ^39^	Wip1 suppresses liver regeneration through dephosphorylation of mTOR.	Wip1 inhibition can activate the mTORC1 pathway to promote proliferation in situations in which liver regeneration is critical.
Kaji *et al.* (2016) ^40^	DNMT1 loss in hepatocytes causes global hypomethylation, initiating senescence and a gradual loss of regenerative capability.	This triggers DNA damage and DNA damage response in hepatocytes, leading to HPC expansion and differentiation to hepatocytes.
Pauta *et al*. (2016) ^41^	The serine-threonine kinases Akt1 and Akt2 phosphorylate and inactivate the transcription factor FoxO1.	Double KO mice have impaired liver regeneration.
Sun *et al*. (2016) ^42^	Loss of Arid1A, a SWI/SNF chromatin remodeling complex component, enhances regeneration by blocking chromatin access to transcription factors that promote differentiation and inhibit proliferation (for example, C/EBPα).	Transient epigenetic reprogramming via Arid1A inhibition may boost regeneration after severe injury.
Swiderska-Syn *et al*. (2016) ^43^	Disrupting Hedgehog signaling in myofibroblasts inhibits regeneration after PH and is associated with a loss of paracrine signals that upregulate Yap1- and Hedgehog-related transcription factors in hepatocytes.	This demonstrates a critical role of paracrine stromal-to- epithelial signaling for effective regeneration.

These are rodent studies unless stated otherwise. C/EBP, CCAAT-enhancer-binding protein; DNMT1, DNA methyltransferase 1; HCC, hepatocellular carcinoma; HDAC, histone deacetylase; HPC, hepatic progenitor cell; InsP3, inositol-1,4,5-trisphosphate; KO, knockout; mTOR, mammalian target of rapamycin; mTORC1, mammalian target of rapamycin complex 1; NF-κB, nuclear factor kappa B; Nrf2, nuclear factor erythroid 2-related factor 2; PH, partial hepatectomy; piRNA, P-element-induced wimpy testis (PIWI)-interacting RNA; SWI/SNF, SWItch/sucrose non-fermentable; TEAD, TEA domain family transcription factors; Wip1, Wild-type p53-induced phosphatase 1; Yap, Yes-associated protein.

The observations of the Nusse group of a largely diploid stem/progenitor hepatocyte population in the immediate pericentral region ^14^, if confirmed, raise a number of questions regarding regeneration after toxic injury and the role of this population in liver cancer histogenesis. Toxins such as CCl _4_ destroy the pericentral regions, yet the liver regenerates perfectly well from other hepatocytes, but can Axin2-positive hepatocytes be re-created after such injury and can their diploid status be re-established from polyploid hepatocytes? Cytokinesis without DNA synthesis in binucleated hepatocytes with diploid nuclei would be one way.

Perivenous diploid hepatocytes are also found in human liver ^3^, so are there implications for liver pathology (for example, in the origins of hepatocellular carcinoma [HCC])? HCCs have similarities with Axin2-positive hepatocytes; many human HCCs are composed of diploid or near-diploid (aneuploid) hepatocytes ^44– 46^, and in childhood hepatoblastoma a small cell (diploid?) undifferentiated histology carries a poor prognosis ^47^. Additionally, expression of nuclear β-catenin and GS can be found in HCCs ^48^, and mutations in the *APC*, *AXIN1*, *AXIN2*, and *CTNNB1*/β-catenin genes are common in human HCCs ^49, 50^.

## Periportal/portal stem cell niche(s)

**Figure 2.**
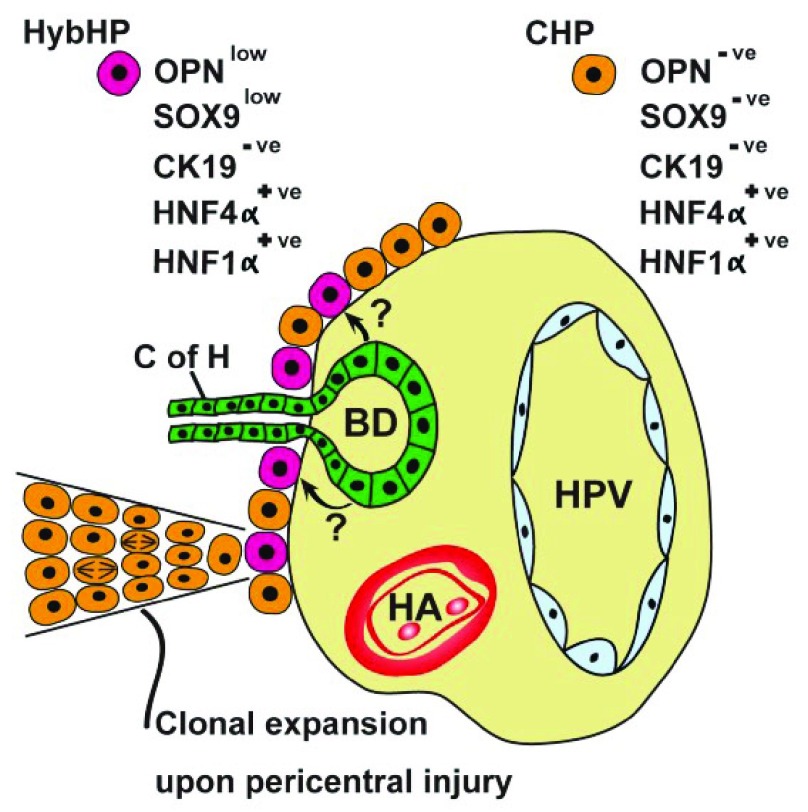
A periportal stem cell niche. A subpopulation of periportal hepatocytes (HybHPs) in intimate contact with the biliary epithelium can clonally expand upon liver injury, migrating towards the central veins. See section entitled ‘periportal/portal stem cell niche(s)’ and 51 for further details. Arrows indicate possible paracrine influences of biliary epithelium upon HybHPs. BD, bile duct; CHP, conventional hepatocyte; C of H, canal of Hering; HA, hepatic artery; HPV, hepatic portal vein; HybHP, hybrid hepatocyte.

If normal homeostatic renewal is fed by pericentral hepatocytes, what happens after toxin-induced injury when pericentral cell death invariably features? HPCs derived from the canals of Hering can be mobilized when hepatocyte regeneration is compromised (see below), but another recent murine study suggests that chronic chemical damage induces clonal expansion of ‘hybrid hepatocytes’ (HybHPs) ( Figure 2), so-called because they express the hepatic fate-determining transcription factor HNF4α, but also low levels of bile duct-enriched genes such as *Sox9* and *OPN*, but no expression of the biliary cytokeratin CK19 ^51^. These HybHPs comprised only 5% of all hepatocytes and exhibited a transcriptome unique from conventional hepatocytes and bile duct epithelia. In the mouse, these hepatocytes were found abutting the limiting plate, often in close association with the terminal branches of bile ducts. Lineage tracing found that HybHPs gave rise to only about 9% of hepatocytes 4 weeks after a single dose of CCl _4_ but contributed to two-thirds of hepatocytes after repeated CCl _4_ injections in *Sox9-Cre ^ERT^;R26R ^YFP^* mice within 12 weeks of tamoxifen treatment. The descendants of HybHPs extended from the portal tracts to the CVs, where they expressed GS, indicative of the new cells adopting the correct metabolic zonation. In the MUP-uPA (major urinary protein-urokinase-type plasminogen activator) mouse, where widespread DNA damage occurs because of endoplasmic stress caused by over-expression of uPA, HybHP progeny could reach the pericentral region within 5 to 6 weeks. Cholestatic injury induced many HybHPs to transdifferentiate to duct cells, strongly expressing Sox9, CK19, and OPN; so perhaps some ductular reactions are derived from hepatocytes? Sox9 and OPN are also expressed in some human periportal hepatocytes, suggesting that HybHPs are also found here. HybHPs were also compared with other hepatocytes for their ability to re-populate the fumarylacetoacetate hydrolase ( *Fah ^-^* ^/ *-*^) null mouse, a model of hereditary tyrosinemia. HybHPs formed Fah ^+^ colonies 2.5-fold larger than those formed from conventional hepatocytes; moreover, mouse survival was vastly superior with HybHP transplantation compared with conventional hepatocyte transplantation. Finally, in three models of HCC formation, neither HPCs nor HybHPs contributed to tumor formation. This inability is probably due to the low expression of drug-metabolizing enzymes by these cells and, of course, is the reason why such cells are immune from the effects of chemicals that cause pericentral damage.

It is important to note that the rate of re-population by so-called HybHPs across the lobule (measured in weeks) was far too low to account for the restitution of liver structure (measured in days) after the likes of a single exposure to CCl _4_; thus, regenerative growth most likely involves all surviving hepatocytes. Moreover, if the pericentral niche is re-established after such an insult, is there a bidirectional flux of hepatocytes? Interestingly, hybrid/transitional/intermediate hepatocytes have also been noted by others in the immediate periportal area, but the relationship of these hepatocytes to HybHPs is unclear. For example, Isse *et al*. ^52^ reported on the presence of CK19 ^*–*^, HNF1β ^+^, HNF4α ^+^ hepatocytes in human liver, whereas the Reid group ^53^ observed hepatoblasts expressing α-fetoprotein, intercellular cell adhesion molecule-1 (ICAM-1), albumin, and membranous epithelial cell adhesion molecule (EpCAM) in the mouse liver. These hepatoblasts appeared to be tethered to the canals of Hering and, importantly, expanded in number during regenerative responses, just like HybHPs; so are these one and the same cells?

HPCs, named oval cells in rodents, give rise to the so-called ductular reaction that is observed in many forms of chronic liver injury. These cells undoubtedly have a biliary phenotype, but their cell of origin is in question. Additionally, there has been considerable debate as to the ability of oval cells/HPCs to generate meaningful numbers of hepatocytes. Ductular reactions probably arise from small intraportal bile ducts and from the canals of Hering, conduits that connect bile canaliculi to intraportal bile ducts ^54^. In both rats ^55^ and mice ^56^, retrograde ink injections via the extrahepatic bile duct have demonstrated continuity between the intraportal bile ducts and ductular reactions. On the other hand, it has been suggested that some ductular reactions arise from the transdifferentiation of hepatocytes; for example, mice fed diethoxycarbonyl-1,4-dihydrocollidine (DDC) generate ductular cells from albumin-positive hepatocytes ^57^, seemingly reprogramming that is dependent on Notch signaling ^58^, but it has been proposed that the ability of hepatocytes to reversibly transdifferentiate to ductular cells is a mechanism to escape injury and expand before redifferentiating to hepatocytes ^59^. The latter study used the *Fah* ^-/-^ mouse that generates a severe form of tyrosinemia combined with DDC that causes bile duct destruction and cholestasis, circumstances so severe that some commentators ^60^ have questioned the relevance of the findings to normal liver regeneration. However, there is evidence that some cholangiocarcinomas, originally considered to be derived from ductular epithelia, actually can have their origins in hepatocytes that have undergone ductular metaplasia ^61, 62^.

Doubts have been cast on the ability of HPCs to make a physiologically useful contribution to hepatocyte replacement *in vivo* ^63– 65^. Much of this skepticism seems to have arisen from the use of mechanistically different oval cell induction models. One classic model involves feeding rats 2-acetylaminofluorene (2-AAF), a compound that is metabolized by hepatocytes to form DNA adducts that render hepatocytes unable to proliferate in response to 2/3 PH. This model, in effect, mimics hepatocyte senescence, a seemingly key factor in inducing ductular reactions when chronic liver injury occurs. With this model, a number of studies suggest that oval cells can undergo hepatocytic differentiation ^55, 66– 68^; moreover, studies of human liver cirrhosis indicate that HPCs also give rise to regenerative hepatocyte nodules ^69, 70^. A lack of any significant contribution by ductular cells to hepatocytes in several injury models could be due to the fact that a complete hepatocyte-senescence-like state is not achieved; Yanger *et al*. ^64^ used DDC, which targets the biliary epithelium, and a choline-deficient, ethionine-supplemented (CDE) diet that induces a fatty liver; the latter model was also used by Schaub *et al*. ^65^.

**Figure 3.**
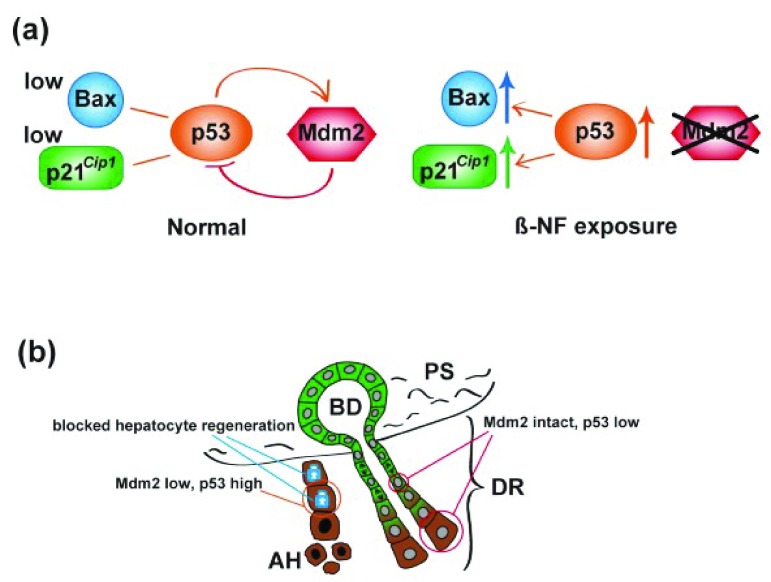
A model for major hepatic progenitor cell activation and hepatocytic differentiation. ( **a**) Molecular mechanism: β-NF injection leads to hepatocyte-specific deletion of *mdm2*, in turn reducing proteasomal destruction of p53 and upregulation of p53 targets p21 and Bax. ( **b**) Cartoon of histological consequences: hepatocytes (brown cytoplasm) undergo cell cycle arrest and apoptosis as a consequence of upregulation of p21 and Bax, respectively. The ductular (green cytoplasm) reaction (DR) is activated, leading to columns of proliferating cells migrating into the parenchyma and eventually differentiating to hepatocytes. See section entitled ‘periportal/portal stem cell niche(s)’ and 71 for further details. β-NF; β-Naphthoflavone; AH, apoptotic hepatocyte; BD, bile duct; PS, portal space.

A further study has now shown how crucial the blockade of hepatocyte regeneration is to HPC activation and differentiation, with complete re-population of the injured liver by nascent hepatocytes under the CDE diet regime ^71^! Lu *et al*. ^71^ inactivated the *Mdm2* gene in hepatocytes, promoting a rise in p53 and increased expression of p21 ^*Cip1*^ and Bax. The resultant hepatocyte senescence and apoptosis led to the almost complete re-population of the liver by HPC-derived hepatocytes ( Figure 3). A detailed account of the molecular regulation of the ductular reaction is beyond the scope of this review, but important molecules include the cytokine tumor necrosis factor-like weak inducer of apoptosis (TWEAK) ^72^, neural cell adhesion molecule (NCAM) ^73^, polycomb-group proteins ^74^, connective tissue growth factor (CTGF) ^75^, Notch ligands ^76^, and integrin αvβ6-dependent transforming growth factor-beta 1 (TGFβ1) activation ^77^.

This review has illustrated some recent observations regarding liver regeneration. We suggest that the functional significance of HPCs is very much dependent on the liver injury model used and that hepatocyte senescence in the face of injury is a major driver for HPC expansion and differentiation. Additionally, we have highlighted studies identifying new stem/progenitor hepatocytes at opposite ends of the PV-CV axis: at the portal rim, clonogenic HybHPs are activated only in response to damage, whereas pericentral hepatocytes are proposed to be responsible for normal turnover, moving in the opposite direction. A recent article has failed to confirm the presence of pericentral stem/progenitor cells ^19^. The kinetics of clonogenic expansion from HybHPs is relatively slow, but since midzonal/centrilobular necrosis induced by the likes of a single injection of CCl _4_ can be repaired within a few days, hepatocyte self-duplication from other surviving hepatocytes must occur, undoubtedly as the dominant mechanism. On the other hand, HybHPs may have superior potential over conventional hepatocytes as a cell therapy. What is certain is that recent studies have revived the debate on the nature of liver regeneration on two counts: firstly, whether the liver conforms to a stem cell and lineage system and, secondly, whether hepatocytes do migrate (stream) and, if so, which way? Or do they migrate both ways?
